# SPARC Overexpression Promotes Liver Cancer Cell Proliferation and Tumor Growth

**DOI:** 10.3389/fmolb.2021.775743

**Published:** 2021-11-29

**Authors:** Zhao-wei Gao, Chong Liu, Lan Yang, Ting He, Xia-nan Wu, Hui-zhong Zhang, Ke Dong

**Affiliations:** Department of Clinical Laboratory, Tangdu Hospital, Air Force Medical University, xi’an, China

**Keywords:** SPARC, liver cancer, proliferation, tumor growth, bioinformatics

## Abstract

**Background:** Secreted protein acidic and rich in cysteine (SPARC) plays an important role in cancer development. The roles of SPARC in the liver hepatocellular carcinoma (LIHC) are unclear.

**Methods:** GEPIA2 and UALCAN were used to analyze the SPARC mRNA expression levels in LIHC based on the TCGA database. The GEO database was used to verify the analysis results. Immunohistochemical (IHC) analysis was used to investigate the SPARC protein levels in LIHC tissues. The Kaplan–Meier (KM) plotter was used to analyze the correlation between SPARC and prognosis. The serum SPARC levels were measured by ELISA. CCK8 and murine xenograft models were used to investigate the effect of SPARC on the liver cancer growth *in vitro* and *in vivo*. SPARC-correlated genes were screened by LinkedOmics.

**Results:** Based on the TCGA and GEO databases, the analysis showed that the SPARC mRNA expression levels were increased in tumor tissues and peripheral blood mononuclear cell (PBMC) from LIHC compared to normal controls. The IHC analysis showed an increased level of SPARC in LIHC tissues compared to adjacent non-tumor tissues. However, we found that the serum SPARC levels were lower in LIHC than those in healthy controls. The KM plotter showed that there was no significant correlation between the SPARC mRNA levels and overall survival. However, in sorafenib-treated LIHC patients, the high SPARC expression predicts favorable prognosis. Furthermore, the endogenous SPARC overexpression promotes liver cancer cell proliferation *in vitro* and tumor growth *in vivo*, while there was no significant effect of exogenous SPARC treatment on liver cancer cell proliferation. Function enrichment analysis of SPARC-correlated genes indicated a critical role of interaction with an extracellular matrix in SPARC-promoting cancer cell proliferation.

**Conclusion:** SPARC mRNAs were increased in LIHC tumor tissues, and SPARC overexpression may promote the liver cancer growth. Further studies are needed to clarify the potential prognostic value of SPARC, both in tissues and in circulation.

## Introduction

According to the Global Cancer Observatory (GLOBOCAN) data from 2020 (Sung et al., 2021), primary liver cancer is currently the sixth most commonly diagnosed cancer (4.7%) and the third cause of cancer death (8.3%) worldwide. Notably, the incidence rate (6.3%) and the mortality rate (10.5%) of primary liver cancer are higher in males. Liver hepatocellular carcinoma (LIHC) is the most common subtype (75–85%) of primary liver cancer (Sung et al., 2021). The risk factors for LIHC include hepatitis virus (HBV and HCV) infection, aflatoxin, excessive drinking, and obesity (Sung et al., 2021). However, the molecular mechanism involvement in LIHC progression is still unclear.

SPARC (secreted protein acidic and cysteine rich; Gene ID: 6678) encodes a cysteine-rich acidic matrix-associated protein, which belongs to the extracellular matrix protein family. SPARC is required for the collagen in bone to become calcified, and is also involved in regulating the cross talk between the extracellular matrix and cell biological behavior, such as cell shape, proliferation, and migration (Bornstein and Sage, 2002). Thus, SPARC has been found to play an important role in tumor progression (Tai and Tang, 2008). First, the SPARC expression has been found to be either increased or decreased in different tumor types. For example, the higher levels of SPARC expression have been reported in breast cancer (Lien et al., 2007) and glioblastomas (Rempel et al., 1998), while the lower levels of SPARC expression have been found in ovarian (Yiu et al., 2001), colorectal (Yang et al., 2007; Cheetham et al., 2008), and pancreatic cancers (Puolakkainen et al., 2004). And moreover, the functions of SPARC in different cancers are controversial. The studies have shown that SPARC overexpression could act as a tumor suppressor gene, which inhibits cancer proliferation, invasion, and angiogenesis in certain types of cancer, including gastric cancer (Zhang et al., 2012), medulloblastoma (Bhoopathi et al., 2010; Bhoopathi et al., 2011), ovarian cancer (Said and Motamed, 2005), and prostate cancer (Wong et al., 2007). In contrast, the high SPARC expression showed a correlation with the highly aggressive tumor phenotype, through promoting tumor cell survival, proliferation, and invasion, such as glioma (Shi et al., 2004) and melanoma (Ledda et al., 1997; Alvarez et al., 2005).

The roles of SPARC in LIHC are unclear. In this study, we first analyzed the difference of SPARC expression between LIHC and normal controls, and then investigated the effects of SPARC overexpression on liver cancer cell proliferation *in vitro* and *in vivo*. The function enrichment analysis of SPARC-correlated genes was used to explore the potential mechanism of SPARC in LIHC. This study provided evidence for the involvement of SPARC in LIHC progression.

## Material and Method

### SPARC Expression-Level Analysis

The GEPIA2 (Tang et al., 2019), UALCAN (Chandrashekar et al., 2017), and GEO databases were used to analyze the difference of SPARC expression levels between LIHC tumor and non-tumor tissues. By using the “Expression DIY” module of GEPIA2 and the “TCGA Analysis” module of UALCAN, differential expression of SPARC mRNA was obtained. UALCAN was also used to analyze the promoter methylation level of SPARC. GSE14323 (Levy et al., 2016), GSE14520 (Roessler et al., 2010; Wang et al., 2021), and GSE121248 (Wang et al., 2007) datasets contain the gene expression profiling of LIHC tumor and their matched normal adjacent tissues, which were used to validate the analysis results based on GEPIA2 and UALCAN. GSE58208 and GSE49515 (Shi et al., 2014) datasets contain the gene expression profiling of PBMC from LIHC patients and healthy subjects, which were used to evaluate the difference of SPARC expression in PBMC.

### Tissue Microarrays and Immunohistochemical Analysis

Formalin-fixed tissue microarray which contained 48 LIHC and 48 matched adjacent controls (D097Lv01, http://www.bioaitech.com/chip-design/13c0de19d7e94d4a99952af585089847.html) was purchased from Bioaitech (Xi’an, China). A 1:200 dilution of anti-SPARC mAb (D10F10, Cell Signaling Technology) was used as the primary antibody. PANNORAMIC and CaseViewer 2.4 software (3DHISTECH, Hungary) were used for image acquisition and analysis. IHC analysis was conducted by two independent pathologists. The intensity was scored as 0 (negative), 1 (weak), 2 (moderate), and 3 (strong). The extent of positive cells less than 5% was scored as 0, 6%–25% was scored as 1, 26%–50% was 2, 51%–75% was 3, and >75% was 4. The final score was determined by multiplying the intensity and extent positivity scores. The detail information of tissue microarrays and IHC scores is available in Supplementary Table S1.

### Kaplan–Meier Plotter Analysis

The KM plotter bioinformatic tools (Menyhárt et al., 2018) were used to evaluate the potential prognostic value of SPARC expression in LIHC patients. We chose the “auto select best cutoff” model, which means that all possible cutoff values were computed and the best performing threshold was used as a cutoff.

### Serum SPARC Level Detection

Ethical approval was obtained from the Ethics Committee of Tangdu Hospital, Fourth Military Medical University (TDLL-202106-04). Informed consent was exempted for this study. Serum from 39 LIHC patients and 32 healthy controls was collected. PBMC from 10 LIHC patients and 10 healthy individuals was collected. PBMC was obtained by using Ficoll separation according to the operation manual (Dakewe Biotech, China).

Serum SPARC levels were measured by the ELISA assay (ELH-SPARC-1, RayBiotech, United States). In brief, 100 μl diluted serum were added into test wells and incubated for 2.5 h at room temperature. Then, the solution was discarded and washed 4 times, and 100 μl biotinylated antibody was added and incubated for 1 h. After washing 4 times, streptavidin was added and incubated for 45 min. The TMB reagent was added into wells, incubated for 30 min, followed by adding stop solution and absorbance determination at 450 nm.

### Cell Culture and Cell Codel Construction

HepG2 cells were cultured in the DMEM (Gibco, United States) plus 10% heat-inactivated fetal bovine serum (FBS, Sijiqing Biotec, China) at 37°C with 5% CO_2_ in a humidified incubator. The SPARC coding gene was cloned into the pcDNA3.1 + expression vector. Then the SPARC/pcDNA3.1+ and control plasmids were transfected into HepG2 cells using Lipofectamine 2000 (Invitrogen, United States) according to the manufacturer’s instruction. Briefly, HepG2 cells were seeded in 6-well plate and cultured until 70–80% confluence. Premixed lipofection and plasmid DNA (10 μl: 4 μg) were added into the wells and incubated for 24 h. The stable cell models were screened by G418. The expression levels of SPARC in cell models were investigated by the quantitative real-time PCR (qRT-PCR). The primers used for qRT-PCR are listed in Table 1.

**TABLE 1:** Primer sequences.

Gene	Forward (5′-3′)	Reverse (5′-3′)
SPARC	GCA​GAG​GAA​ACC​GAA​GAG​GAG	AGT​GGC​AGG​AAG​AGT​CGA​AGG
GAPDH	CCA​CAT​CGC​TCA​GAC​ACC​AT	GGC​AAC​AAT​ATC​CAC​TTT​ACC​AGA​GT

### Cell Proliferation Detection

Cell proliferation was investigated by the CCK8 assay. In short, cells were seeded into 96-cell plate at a density of 2 × 10^3^ cells/well, with six replicate wells per group. After 24 and 48h, a 10 µl CCK8 reagent (KeyGEN BioTECH, Nanjing, China) was added, followed by incubation for 2 h. The absorbance value was measured at 450 nm by using the microplate reader (BIO-TEK Epoch, United States).

### Cell Cycle and Apoptosis Detection

Flow cytometry (FCM) was used to analyze the cell cycle and apoptosis. The cell cycle was analyzed by ethanol-fixed cells stained with propidium iodide (PI) in buffer containing RNase A. The DNA content was assessed by using the FACS calibur flow cytometer (BD Biosciences). Cell apoptosis was detected using the annexin V–FITC apoptosis detection kit (KeyGEN BioTECH, Nanjing, China). Briefly, cells were collected and washed with PBS twice, and stained for 15 min at room temperature with annexin V-FITC and (PI). Following that, cell apoptosis was examined using FACS calibur. The percentage of apoptotic cells was calculated with CellQuest 6.0 (BD Biosciences).

### Murine Xenograft Models

All procedures of animal experiments were processed according to animal welfare guidelines approved by Air Force Medical University’s Institutional Animal Care and Use Committee. Male BALB/c nude mice were used. The effect of high SPARC expression on tumor growth *in vivo* was assessed by subcutaneous injection of 2×10^6^ HepG2^*SPARC*^ or HepG2^*NC*^ cells into a BALB/c nude mouse. Nude mice were purchased from Animal Experiment Center, Fourth Military Medical University (age: 6–8 weeks; body weight: 20–25 g). Tumor size was measured every 2 days with a vernier caliper. The volume of tumor was calculated according to the formula: *a*×*b* ^2^ × 0.5 (*a*, largest diameter and *b*, perpendicular diameter).

### Function Enrichment Analysis of SPARC Correlated Genes

The gene set which was positively or negatively correlated to SPARC in LIHC was screened by the LinkedOmics analysis tool (http://www.linkedomics.org) based on TCGA data (Vasaikar et al., 2018). The gene ontology (GO) and KEGG pathway analyses were performed by DAVID Bioinformatics Resources (https://david.ncifcrf.gov/tools.jsp) (Huang et al., 2009). R Bubble diagrams were used for visualization of the enriched GO term and KEGG pathway.

### Statistical Analysis

Student’s t-test was used to analyze the difference of IHC scores between tumor tissues and controls. Student’s t-test was used to analyze the difference of SPARC expression between the LIHC and control, both in tissues and in PBMC. Serum SPARC levels were expressed as the median (inter quartile range, IQR). Wilcoxon’s test was used to analyze the difference of serum SPARC levels between the LIHC and healthy controls. Student’s t-test was used to analyze the difference of the cell proliferation rate between different groups. *p* < 0.05 was considered to be statistically significant.

## Results

### SPARC Expression Were Increased in LIHC Tissues

**FIGURE 1:**
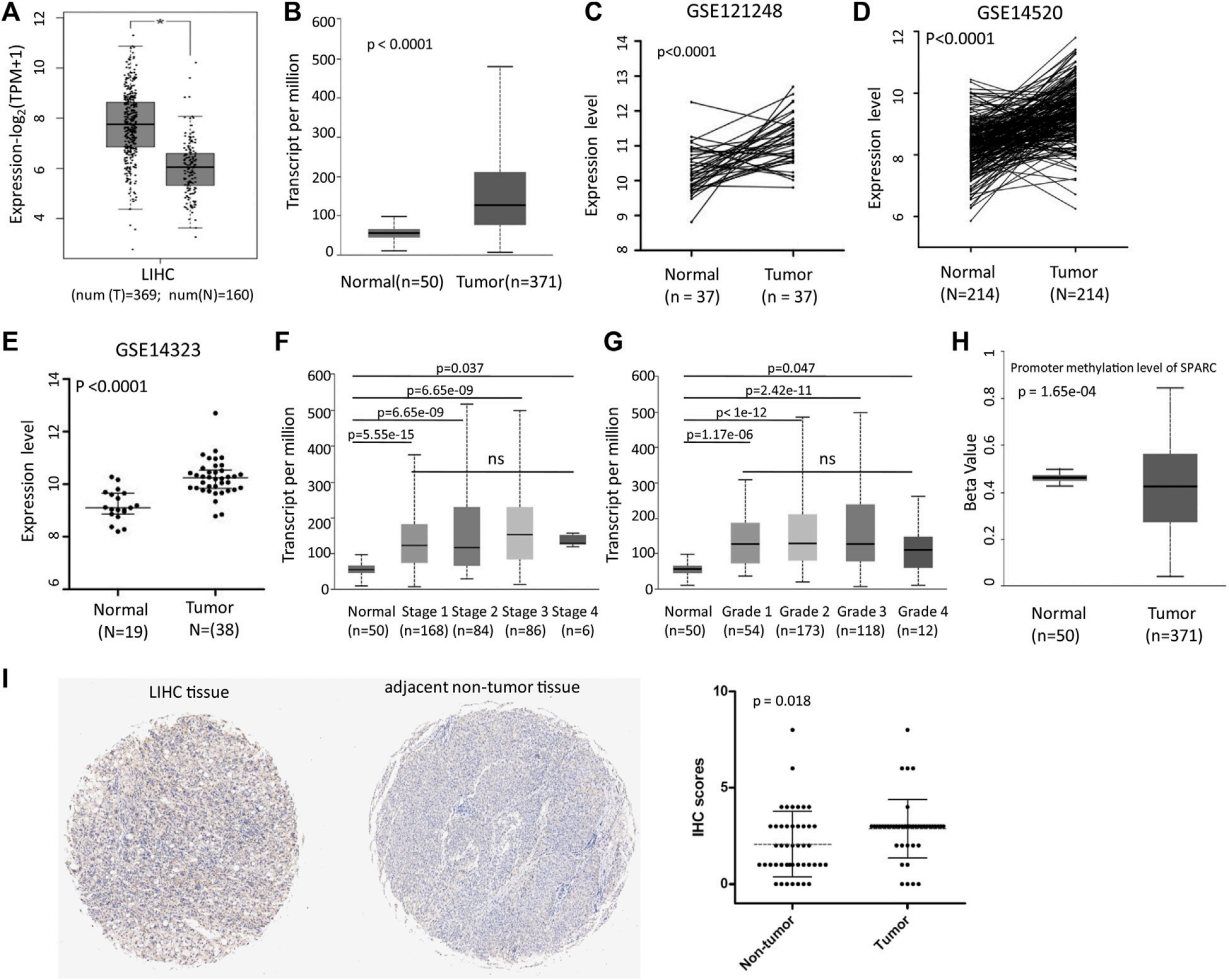
SPARC mRNA expression in LIHC tissues and normal controls. **(A)** Differential SPARC mRNA expression between the LIHC and normal live tissues in the GEPIA2 database. **(B)** Differential SPARC mRNA expression between the LIHC and normal controls in the UALCAN database. **(C–E)** Differential SPARC expression between the LIHC tissues and non-tumor tissues in GSE121248, GSE14520, and GSE14323 datasets. **(F, G)** Boxplot of SPARC expression based on the tumor stage and grade. **(H)** Boxplot of SPARC promoter methylation level. **(I)** IHC analysis of LIHC tissue microarray.

We first analyzed the SPARC expression levels in LIHC patients based on the TCGA and GEO databases. GEPIA2 and UALCAN analyses showed an increased mRNA expression level of SPARC (Figures 1A,B). And moreover, the results were validated in GSE121248, GSE14520, and GSE14323 datasets (Figures 1C–E). These data showed that SPARC expression levels were higher in LIHC tumor tissues than those in control normal tissues. Notably, there was no significance of the SPARC expression between LIHC patients with different stage or grade (Figures 1F,G). And moreover, the UALCAN analysis showed a lower promoter methylation level of SPARC in LIHC than that in normal controls (Figure 1H), which indicated demethylation might contribute to an increased expression of SPARC in LIHC.

Furthermore, the IHC analysis of tissue microarrays was used to investigate the protein expression levels of SPARC in LIHC tissue. The IHC analysis revealed an increased level of the SPARC protein in LIHC tissues compared to adjacent non-tumor tissues (Figure 1I). Taken together, these results demonstrated that SPARC was increased in LIHC tissues.

### The Potential Prognostic Value of SPARC

Furthermore, the relationship between the SPARC expression and prognosis of LIHC patients was analyzed by using the KM plotter. The results showed that there was no significant correlation between the SPARC mRNA expression and LIHC patients’ overall survival (OS) (Supplementary Figure S1A), whether or not the patients were with hepatitis virus (Supplementary Figures S1B,C) or alcohol consumption (Supplementary Figures S1D,E). However, in LIHC patients treated with sorafenib, although the number of patients was small (n = 29), high SPARC expression showed a favorable prognosis (HR = 0.19, logrank *p* = 0.0053; Supplementary Figure.S1F).

### Serum SPARC Levels Decreased in LIHC Patients

**FIGURE 2:**
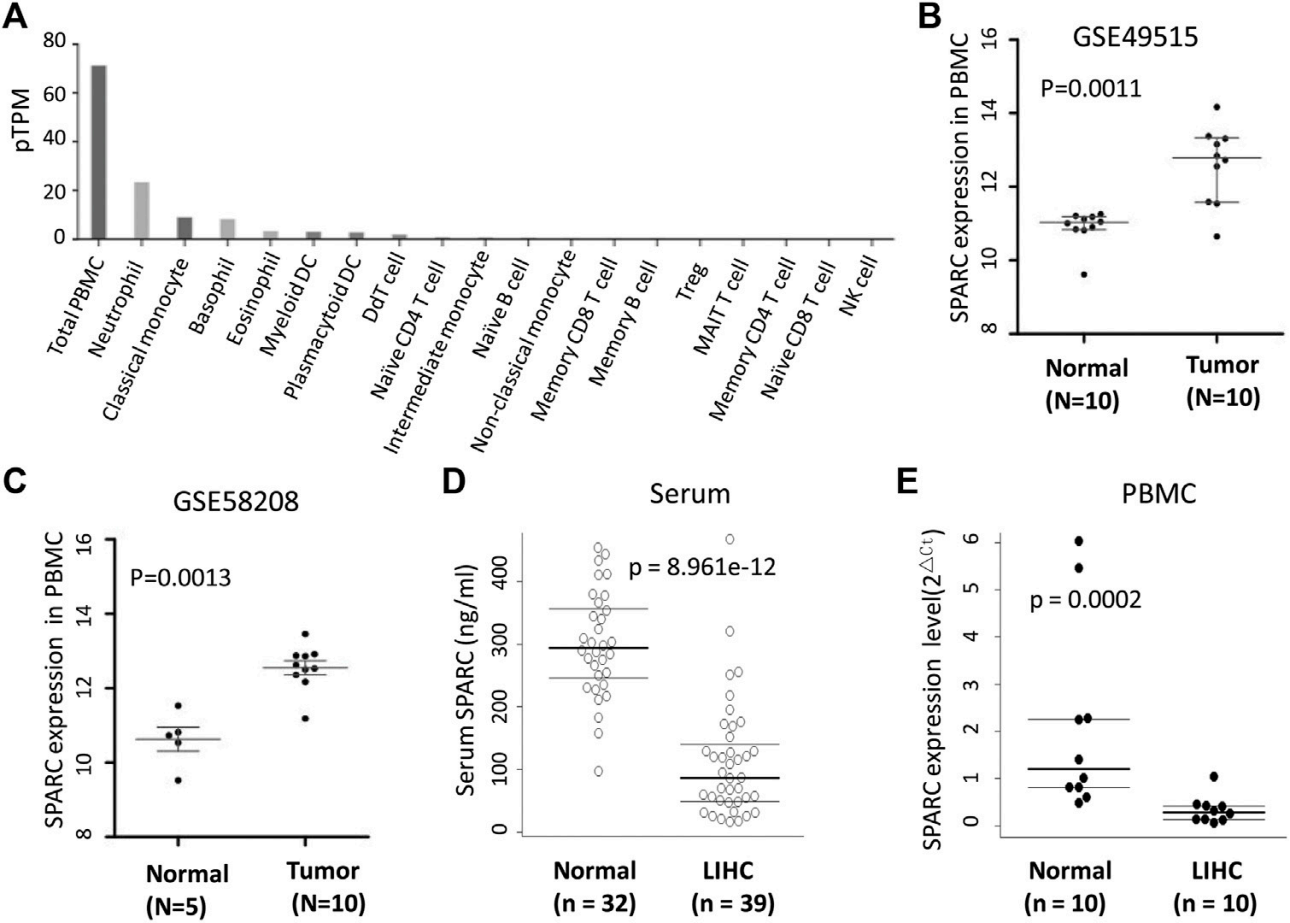
SPARC levels in serum and PBMC from LIHC. **(A)** Human protein atlas (HPA) database showed the SPARC expression profile in blood. **(B, C)** Differential SPARC mRNA expression in PBMC between LIHC and normal controls in GSE49515 and GSE58208 datasets. **(D)** Serum SPARC protein levels in LIHC and healthy controls (HC). **(E)** SPARC mRNA expression levels in PBMC from our sample in this study.

SPARC was expressed in a variety of cells, including PBMC. The human protein atlas (HPA) database showed that SPARC was expressed in total PBMC, monocytes, neutrophil, basophil, eosinophil, *etc* (Figure 2A). Thus, in addition to analyzing the SPARC expression in tumor tissues, we also analyzed the difference of SPARC mRNA expression levels in PBMC from LIHC patients and normal controls based on GSE58208 and GSE49515 datasets. These datasets showed a higher SPARC expression in LIHC’s PBMC than in control’s PBMC (Figures 2B,C).

As SPARC is a secreted protein, we further investigated the SPARC levels in serum from LIHC patients. As shown in Figure 2D, the serum SPARC levels were significantly decreased in LIHC patients compared to healthy controls [86.19 (IQR: 49.16-140.46) vs. 293.53 (IQR: 245.97-356.66) ng/ml, *p* < 0.001)]. Furthermore, by using qRT-PCR, we detected the SPARC mRNA expression in PBMC from LIHC patients and healthy individuals. The results showed a lower expression level of SPARC in LIHC’s PBMC than that in healthy individuals (Figure 2E), which is consistent with our results of serum SPARC detection. However, these data were inconformity with the published data in GSE58208 and GSE49515 datasets.

### Endogenous SPARC Overexpression Promotes Liver Cancer Cell Growth

**FIGURE 3:**
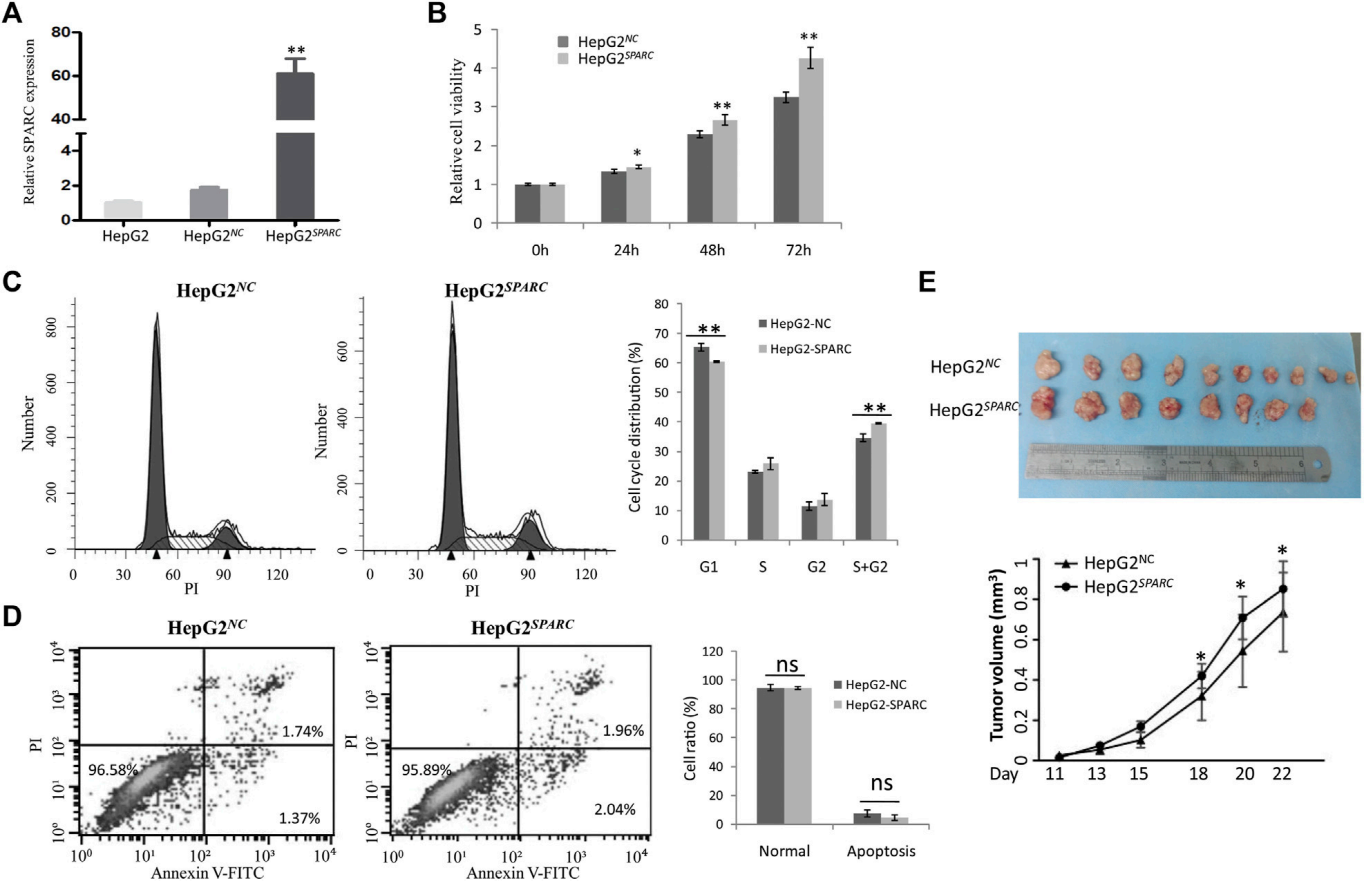
Endogenous SPARC overexpression promote liver cancer cells proliferation and tumor growth. **(A)** qRT-PCR showed the higher SPARC expression level in HepG2^*SPARC*^. **(B)** SPARC overexpression promotes liver cancer cell proliferation. **(C)** FCM showed the effects of SPARC overexpression on cell cycle. **(D)** FCM showed the effects of SPARC overexpression on cell apoptosis. **(E)** SPARC overexpression promotes tumor growth *in vivo*.

We investigated the endogenous SPARC overexpression on HepG2 cell proliferation *in vitro*. First, the SPARC overexpressed cell model was constructed and named as HepG2^*SPARC*^. The higher expression level of SPARC in HepG2^*SPARC*^ than that in HepG2^*NC*^ control cells was identified by qRT-PCR (Figure 3A). Then, by using the CCK8 assay, HepG2^*SPARC*^ cells showed an enhanced proliferation rate compared to HepG2^*NC*^ (Figure 3B). And moreover, the FCM analysis showed that there was a significant decrease of HepG2^*SPARC*^ cells in the G1 phase, while there was an increase in G2 and S phases (Figure 3C). There was no significant difference of cell apoptosis between HepG2^*SPARC*^ and HepG2^*NC*^ cells (Figure 3D).

In addition to the cell experiments *in vitro*, we further investigated the effect of SPARC overexpression on tumor growth *in vivo*. By using nude xenografted models, we found that SPARC overexpression promoted liver cancer growth (Figure 3E). Taken together, these results demonstrated a promoted effect of high SPARC expression on liver cancer growth.

### The Effect of Exogenous SPRAC Treatment on HepG2 Proliferation, Cell Cycle, and Apoptosis

Furthermore, we evaluated the effects of exogenous SPARC treatment on the HepG2 cell biological behavior. The gradient concentration of recombination SPARC protein was added into the cell cultured medium. Then, CCK8 results showed that exogenous SPARC treatment has no significant effect on HepG2 cell proliferation (Supplementary Figure S2A). The FCM analysis results showed that there was no significant effect of exogenous SPARC on cell cycle and apoptosis (Supplementary Figures S2B,C).

### The Function Enrichment Analysis of SPARC-Correlated Genes in LIHC

**FIGURE 4:**
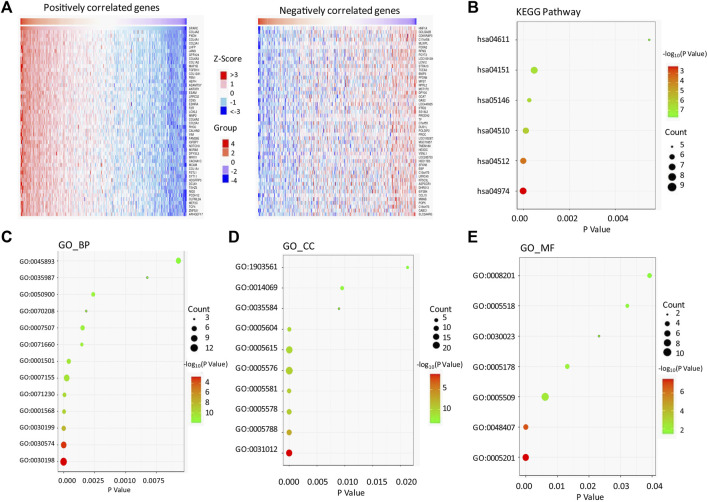
SPARC-associated genes and pathway enrichment in LIHC. **(A)** Heatmaps showing top 50 genes positively correlated **(left)** and top 50 genes negatively correlated with SPARC in LIHC **(right)**. **(B)** Enriched KEGG pathways of SPARC-associated genes. **(C–E)** Enriched GO terms of SPARC-associated genes.

We used the LinkedOmics bioinformatic tool to screen the genes which positively or negatively correlated to SPARC in LIHC (Supplementary Table S2). As shown in the heatmap (Figure 4A), SPARC strongly and positively correlated to the collagen gene family (such as COL4A2, COL4A1, COL3A1, COL6A3, and COL1A2; r > 0.8, *p* < 0.0001), peroxidasin-PXDN (r = 0.893, *p* < 0.0001), and junctional adhesion molecule 3-JAM3 (r = 0.871, *p* < 0.0001). Furthermore, the KEGG pathway and GO enrichment analyses of SPARC association genes were performed (Figures 4B–E; Supplementary Table S2). The results showed that SPARC-correlated gene enrichment in GO_BP (biological process) was mainly related to the extracellular matrix (ECM) organization (GO:0030198), collagen catabolic process (GO:0030574), collagen fibril organization (GO:0030199), and cellular response to amino acid stimulus (GO:0071230) (Figure 4C). GO_CC (cell component) enrichment showed that these genes were mainly expressed in the extracellular matrix (GO:0031012), endoplasmic reticulum lumen (GO:0005788), proteinaceous extracellular matrix (GO:0005578), and extracellular region (GO:0005576) (Figure 4D). The GO_MF (molecular function) analysis showed an enrichment in the extracellular matrix structural constituent (GO:0005201), calcium ion binding (GO:0005509), and platelet-derived growth factor binding (GO:0048407) (Figure 4E). And moreover, the KEGG pathway analysis showed that SPARC-correlated genes were involved in protein digestion and absorption (hsa04974), ECM-receptor interaction (hsa04512), and PI3K-Akt signaling pathway (hsa04151). Taken together, these results indicated a critical role of interaction with the extracellular matrix in SPARC-promoting cancer cell proliferation.

## Discussion

In this study, we aim to evaluate the expression change and potential function of SPARC in LIHC. First, based on TCGA and GEO databases, we found the mRNA expression levels of SPARC were increased in LIHC tumor tissues compared to non-tumor tissues. And moreover, hypomethylation in SPARC promoter might be a cause of its increased expression in LIHC tumor tissues. The IHC analysis of tissue microarray showed an increased level of the SPARC protein in LIHC tumor tissues compared to adjacent non-tumor tissues.

Second, for the prognosis value of SPARC in LIHC patients, the KM plotter analysis showed that there was no significant correlation between SPARC mRNA expression and overall survival of LIHC patients, except in sorafenib-treated patients. The relationship between the higher SPARC mRNA level and favorable prognosis for sorafenib treatment needs to be further verified in a large number of patients. Additionally, the study by Ju MJ et al. suggested that higher expression of SPARC in peritumoral activated hepatic stellate cells is an independent predictor of more early recurrence and poorer survival of LIHC patients (Ju et al., 2009).

SPARC was expressed in a variety of cells, including PBMC. The HPA database showed that SPARC was expressed in total PBMC, monocytes, neutrophil, basophil, eosinophil, *etc*. In this study, we found that the SPARC mRNA expression was lower in PBMC from LIHC patients than that in healthy individuals. As shown in Supplementary Table S3, in our samples, the counts of lymphocyte and eosinophil were significantly lower in LIHC patients than those in healthy controls. Thus, the decreased SPARC mRNA expression in PBMC could be due to the decreased number of lymphocyte and eosinophil. And moreover, serum SPARC protein levels were also lower in LIHC patients than those in healthy individuals. However, the study by Zhang J et al. showed that serum SPARC in LIHC patients was significantly higher than that in healthy controls, while it was significantly lower than that in benign liver disease patients (Zhang et al., 2017a). These inconsistent results need further verification in larger cohorts. The study by Liu et al. showed that lower serum SPARC levels were associated with better prognosis for LIHC patients who have undergone transarterial chemoembolization-TACE (Liu et al., 2020). Thus, serum SPARC might be served as a potential predictor for prognosis of LIHC patients with certain characteristics. Of note, the association between the serum SPARC and prognosis might be conflicting in patients with different cancer types. For example, Akutsu T’s study (Akutsu et al., 2020) showed that digestive tract cancer patients with serum SPARC levels lower than the median level had a significantly higher risk for death than those with higher levels.

Furthermore, our experiments showed a promoted effect of endogenous SPARC overexpression on liver cancer cell proliferation *in vitro* and tumor growth *in vivo*, which might be associated with an increased cell ratio in S and G2 phases. Consistent with this result, the previous study reported that down-regulation of SPARC contributed to the inhibition of proliferation of HuH-7 liver cancer cells (Liu et al., 2020). The study by Jiang et al. found SPARC overexpression promoted tumor growth *via* enhancing the acquisition of stem cell phenotypes in liver cancer cells (Jiang et al., 2019). And moreover, the previous study showed that microRNA-211 could suppress liver cancer cell proliferation and invasion by targeting SPARC, which indicated that SPARC overexpression might drive progression of LIHC (Deng et al., 2016). However, by using a non-alcoholic fatty liver (NAFLD)-related HCC murine model, Onorato et al. (2021) found that the absence of SPARC accelerated HCC development, which is associated with an altered hepatic lipid metabolism. And moreover, the study by Atorrasagasti et al. also showed that SPARC overexpression in hepatocellular carcinoma cells results in a reduced tumorigenicity partially (Atorrasagasti et al., 2010). Taken together, these data demonstrated the important roles of endogenous SPARC during LIHC progression, and SPARC could be a double-edged sword. And moreover, the SPARC-correlated genes were enriched in the biological process which related to ECM organization, collagen catabolic process, collagen fibril organization, *etc*. Thus, the potential mechanism of SPARC in LIHC might be associated with these biological processes. Notably, the gene expression characteristics of patients are only one of the factors that affect their survival. Thus, the function of gene in the biological process is not equal to its prognostic value. For example, the study by Zhang QY et al. showed that FAM46C repressed LIHC cell proliferation (Zhang et al., 2017b). However, the KM plotter showed that there were significant effects of FAM46C on the survival of LIHC patients (HR (95% CI) = 0.74 (0.5-1.09), logrank *p* = 0.13).

In conclusion, we demonstrate that SPARC expression is increased in LIHC patients. And moreover, endogenous SPARC overexpression may promote LIHC progression. Our study showed that SPARC could be a potential therapeutic target for LIHC.

## Data Availability

The original contributions presented in the study are included in the article/ **Supplementary Material** , and further inquiries can be directed to the corresponding author.
