# Supplementary material for: SPARC Overexpression Promotes Liver Cancer Cell Proliferation and Tumor Growth
Source: Front Mol Biosci. 2021 Nov 29;8:775743. doi: 10.3389/fmolb.2021.775743 (PMC8668270; doi:10.3389/fmolb.2021.775743)
Supplement: Supplementary file 3 [file Presentation1.pdf]

SUPPLEMENTARY MATERIAL

Supplementary Table 1. The detail information of LIHC tissue microarrays and IHC scores.

Supplementary Table 2. The detail information for function enrichment analysis of SPARC correlated genes in LIHC.

Supplementary Table 3. The white cell count of LIHC patients and healthy controls.

Supplementary Figure 1 (Image 1). Prognostic value of SPARC in LIHC. (A) KM survival analysis of SPARC in LIHC via KM Plotter. (B, C) KM survival analysis of SPARC in LIHC patients with or without hepatitis virus. (D, E) KM survival analysis of SPARC in LIHC patients with or without alcohol consumption. (F) KM survival analysis of SPARC in LIHC patients with sorafenib treatment.

Supplementary Figure 2 (Image 2). The effect of exogenous SPARC treatment on HepG2 cell biological behavior. (A) There were no significant effect of SPARC treatment on cells proliferation. (B, C) FCM analysis showed that there were no significant effect of SPARC treatment on cell cycle and apoptosis.
