# Supplementary figures and images for: SPARC Overexpression Promotes Liver Cancer Cell Proliferation and Tumor Growth
Source: Front Mol Biosci. 2021 Nov 29;8:775743. doi: 10.3389/fmolb.2021.775743 (PMC8668270; doi:10.3389/fmolb.2021.775743)

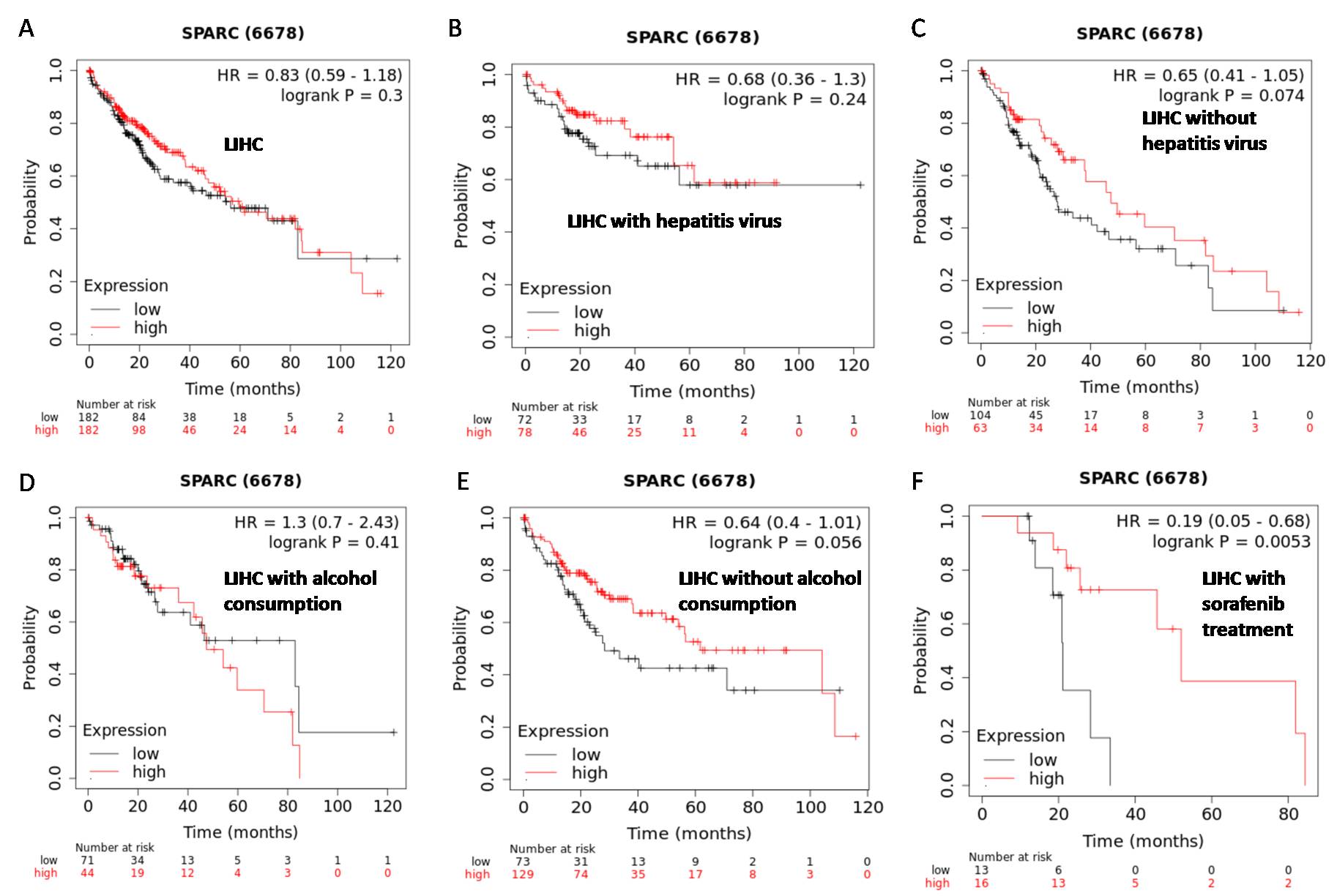

Supplement: Supplementary file 4 [file Image1.JPEG]

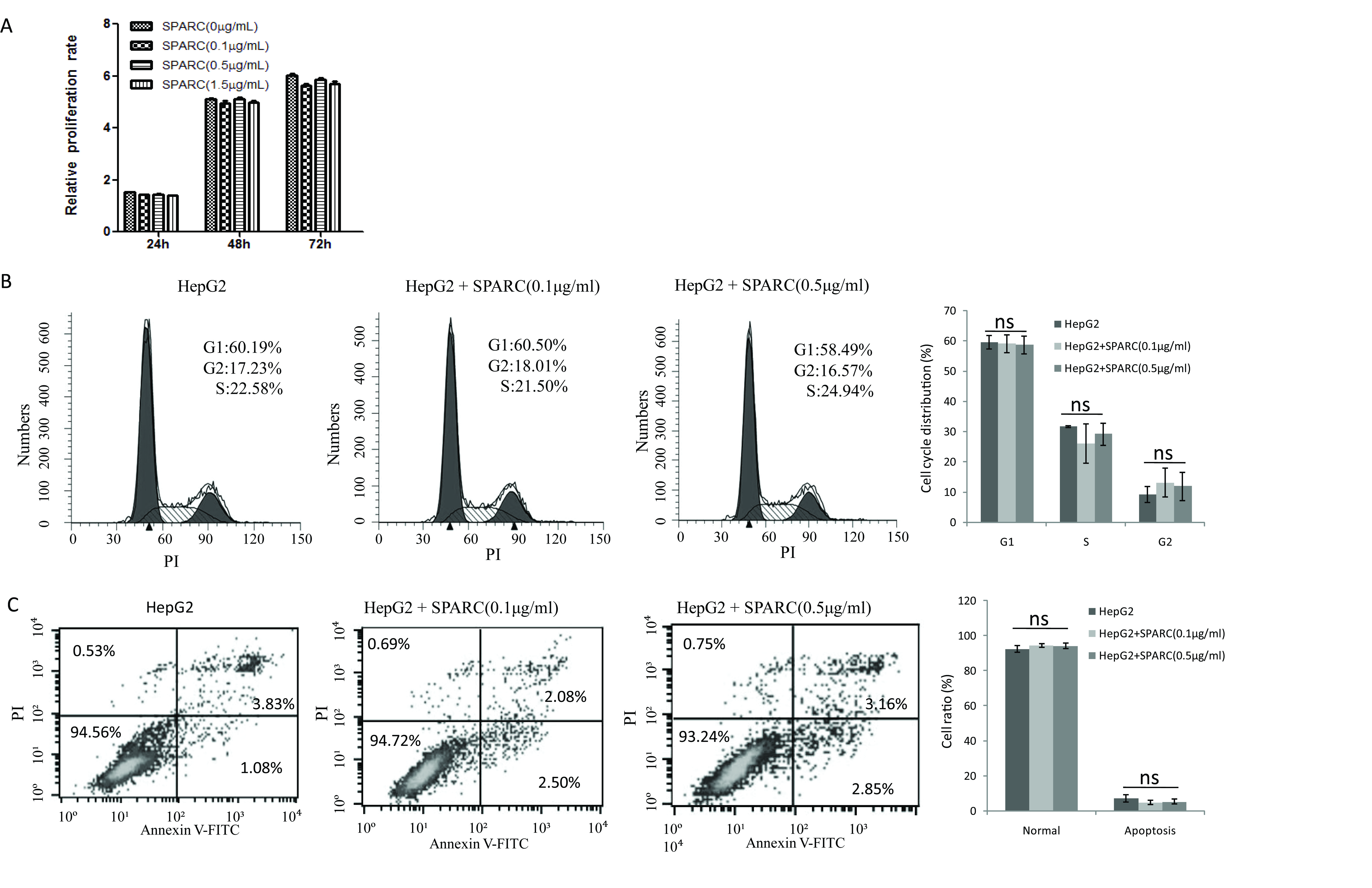

Supplement: Supplementary file 6 [file Image2.JPEG]
